# Measurement for Something as Personal as Dressing is Not Personalized

**DOI:** 10.1093/geroni/igab046.1012

**Published:** 2021-12-17

**Authors:** Lindsay Prizer, Sheryl Zimmerman

**Affiliations:** 1 Emory University, Atlanta, Georgia, United States; 2 Cecil G. Sheps Center for Health Services Research, Chapel Hill, North Carolina, United States

## Abstract

In 2018, the Alzheimer’s Association set forth Dementia Care Practice Recommendations in nine domains, one being support for activities for daily living (e.g., dressing, toileting, eating/nutrition). For example, preservation of dressing independence is important for dignity, autonomy, and to decrease caregiver burden. Measurement is necessary to guide care and assess outcomes related to dressing, but availability of related measures to assess processes, structures, and outcomes of care has not been examined; more so, the extent to which the related measures are person-centered is completely unexplored territory. This session will present a critical assessment of available measures grounded in the Donabedian Model. Of 21 identified measures, 4 assessed dressing alone, 16 included dressing as part of a larger scale, and 1 included dressing as a part of a scale to screen for dementia; none were person-centered. This session will suggest modifications to and need for new measures for person-centered dressing.

